# Image analysis applied to Brillouin images of tissue-mimicking collagen gelatins

**DOI:** 10.1364/BOE.10.001329

**Published:** 2019-02-19

**Authors:** Noemi Correa, Simon Harding, Michelle Bailey, Sophie Brasselet, Francesca Palombo

**Affiliations:** 1 School of Physics and Astronomy, University of Exeter, Stocker Road, EX4 4QL Exeter, UK; 2 Machine Intelligence Ltd, EX20 2JS South Zeal, UK; 3 Aix Marseille Univ, CNRS, Centrale Marseille, Institut Fresnel, F-13013 Marseille, France

## Abstract

Brillouin spectroscopy is an emerging analytical tool in biomedical and biophysical sciences. It probes viscoelasticity through the propagation of thermally induced acoustic waves at gigahertz frequencies. Brillouin light scattering (BLS) measurements have traditionally been performed using multipass Fabry-Pérot interferometers, which have high contrast and resolution, however, as they are scanning spectrometers they often require long acquisition times in poorly scattering media. In the last decade, a new concept of Brillouin spectrometer has emerged, making use of highly angle-dispersive virtually imaged phase array (VIPA) etalons, which enable fast acquisition times for minimally turbid materials, when high contrast is not imperative. The ability to acquire Brillouin spectra rapidly, together with long term system stability, make this system a viable candidate for use in biomedical applications, especially to probe live cells and tissues. While various methods are being developed to improve system contrast and speed, little work has been published discussing the details of imaging data analysis and spectral processing. Here we present a method that we developed for the automated retrieval of Brillouin line shape parameters from imaging data sets acquired with a dual-stage VIPA Brillouin microscope. We applied this method for the first time to BLS measurements of collagen gelatin hydrogels at different hydration levels and cross-linker concentrations. This work demonstrates that it is possible to obtain the relevant information from Brillouin spectra using software for real-time high-accuracy analysis.

## 1. Introduction

Brillouin light scattering (BLS) spectroscopy is an emerging technique in biomedical sciences, biophysics and biophotonics. It is making an impact in these fields as it probes micromechanical properties through an optical high-resolution and contact-free method. In BLS, micromechanical information is obtained via spectral analysis of inelastically scattered light from thermally induced acoustic waves in the GHz range. Longitudinal acoustic modes propagating at a speed of a few km/s give rise to Brillouin peaks in the range 10-20 GHz. The actual frequency shift depends on the stiffness and the linewidth on the attenuation of the acoustic waves in the material [1–3]. Viscoelastic materials such as biopolymers and biomaterials in general exhibit frequency-dependent mechanical responses, and their elastic moduli differ based on the spatial and temporal scale of the technique employed. In these materials, Brillouin measurements giving access to microscale mechanics yield longitudinal elastic moduli of the order of GPa [4,5], whilst traditional quasistatic mechanical testing give Young’s moduli in the MPa range [6]. Whilst this indicates that the techniques probe different forms of elastic modulus, it is also apparent that measurements and molecular dynamics simulations on a nanometre scale also provide Young’s moduli in the GPa range [7].

Traditionally Brillouin spectroscopy has been performed using tandem multipass Fabry-Pérot interferometers, which can achieve very high contrast and spectral resolution [8], the only limitation being the acquisition time of a single spectrum especially in mapping large samples. While coherent methods have been developed to improve speed and sensitivity [9], spontaneous Brillouin techniques are convenient in terms of simplicity of implementation and instrument costs. Here we use spontaneous Brillouin microscopy based on a double-stage cross-axis cascading virtually imaged phase array (VIPA) spectrometer [10,11] capable of acquiring hyperspectral maps of biomedical samples with good contrast and reduced laser power.

Our previous works have demonstrated the application of tandem Fabry-Pérot Brillouin spectroscopy to the studies of fibrous proteins of the extracellular matrix (ECM), providing access to the full elasticity tensor of collagen and elastin fibres [4] and the effects of hydration [6] and purification [5]. We have also applied high-resolution Brillouin microscopy to microbial biofilms [8], histological sections of epithelial tissue in Barrett’s oesophagus [12,13] and to Alzheimer’s brain in a mouse model of amyloidopathy [14,15]. This has demonstrated the versatility and the capability of the technique to spatially map stiffness in correspondence to specific molecular composition in tissues on a microscale.

In the present work, we apply VIPA Brillouin microscopy to collagen gelatin hydrogels at various concentrations and in the presence of a cross-linker, and present a new method to extract the relevant information contained in Brillouin images of biomaterials. Gelatin hydrogels are physical gels derived from bovine skin collagen that are devoid of the complex hierarchical structure (triple helices / fibrils / fibril bundles / fibres) typical of collagen-rich tissues [16], thus providing a homogeneous material for testing. Formalin is commonly used as a fixative for biological samples, to preserve cells and tissues. Here it is added prior to gelation, thus altering the gel structure. Although the method can readily be extended to other specimens, it is applied here to hydrogels that are tissue models with water concentration ranging between 96 and 82 wt. %.

The method that we present here for the first time is capable of automatically analysing Brillouin images, to identify peaks and to apply fit analysis to extract the relevant parameters for viscoelastic characterization. Further, the software automatically corrects for drifts in the pattern of scattered light and other visual distortions, and provides real-time, fully automated data analysis. The ability to complete processing in real time, i.e. faster than the image acquisition rate, will allow for high resolution scanning of large areas without the need to store vast amount of imaging data. Further, algorithm design has been developed with modern high speed parallel processing devices, such as Graphics Processing Units (GPUs), in mind so that the remarkable increase in speed that this type of hardware brings can be applied to the data analysis. The protocol was developed for dual-stage VIPA Brillouin images, however it can be easily adapted to analyse images from a single-stage spectrometer.

This paper is divided into three sections. In Section 2, we present the experimental system and the computer method developed for image acquisition and data processing. We also address issues related to laser drifting and how to correct for those without the need for an additional optical path. In Section 3, we present and discuss the results from image analysis of collagen gelatin hydrogels. Finally, Section 4 draws the conclusion.

## 2. Materials and methods

### 2.1 Collagen gel preparation

Type B 225-Bloom gelatin derived from bovine skin (G9382, Sigma-Aldrich) was combined with appropriate quantities of Milli-Q water to prepare hydrogels of 4 to 18 wt. % gelatin giving a water concentration of 96 to 82 wt. %. Gelatin powder was dissolved in water at 55–65 °C under stirring for 60 minutes, resulting in a clear solution for all concentrations.

Gels containing the cross-linker were prepared using the same protocol, with the addition of formalin (37 wt. % formaldehyde solution, Sigma-Aldrich) when the gel reached 50°C. Hydrogels were prepared with concentrations of formalin in the range 1 to 16 wt. %, with water content adjusted to maintain a constant 10 wt. % gelatin concentration. Gels were poured into moulds at 40°C and covered with Parafilm to prevent drying, then left to cool at room temperature. Approximately 24 hours after gelation, a small piece of gelatin from each mould was transferred to a round glass coverslip (Biochrom, 0.17mm thickness) in an Attofluor cell chamber (Life Technologies) for microscopy.

### 2.2 Brillouin microscopy

**Fig. 1:**
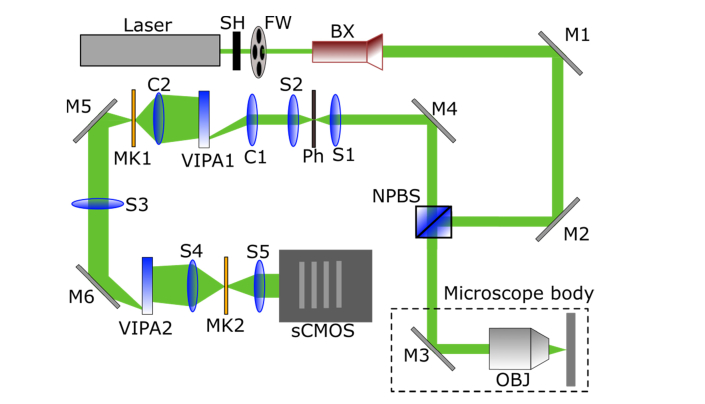
Schematic diagram of the VIPA Brillouin microscope system. Acronyms denote SH: shutter; FW: neutral density filter wheel; BX: 10x beam expander; M1–6: mirrors; NPBS: (10:90) non-polarising beam splitter; C1–2: cylindrical lenses; S1–5: spherical lenses; MK1–2: optical masks.

The Brillouin microspectroscopy system comprised a lab-built double-stage VIPA spectrometer of the type previously presented [17], coupled to an Olympus IX73 two-deck inverted microscope with epi-configuration. A schematic diagram of the apparatus is shown in Fig. 1

.

The light source employed was a single-frequency DPSS laser (Cobolt Samba) operating at 532nm, with 300mW output power and <1 MHz spectral linewidth. The laser beam passed through a shutter and a motorized neutral density filter wheel (Thorlabs) before going through a 10x beam expander (Thorlabs), and then through a (10:90) non-polarising beam splitter (Thorlabs) at the entrance of the microscope. Within the microscope frame, a Chroma sputtered enhanced silver reflective mirror (R ~99.6% at 530nm) was used to direct the laser beam towards the objective. A 60x, 1.20 NA water-immersion objective (Olympus UPlanApo) was used for the Brillouin measurements. The theoretical spatial resolution was: *x*, *y* ~270nm and *z* ~1μm. Backscattered light from the sample was collected using the same objective and directed back to the beam splitter. The transmitted light was collected by a lens (Thorlabs) and focused into a 75μm pinhole (Thorlabs). The filtered light was collimated and directed towards the VIPA spectrometer. This consisted of two cylindrical lenses (C1/C2, Thorlabs, f = 200mm), two spherical lenses (S1/S2, Thorlabs, f = 200mm) and two VIPA etalons (LightMachinery, 30GHz FSR). Two *xy* slits (OptoSigma) were employed to select a dispersion order by the VIPAs and to reject any unwanted stray light through the spectrometer. The spectral resolution was ~0.6 GHz. The detector was an air-cooled sCMOS camera (Andor Zyla 4.2P-USB3), with 13μm x 13μm pixel size. Exposure times at the camera vary between 1 and 4 s, depending on the sample being tested. Laser power at the sample was in the range 3–5 mW. Two PI microscope stages (PILine xy stage system, 100mm x 75mm travel range; PInano z piezo well plate scanner system, 220μm travel range) were used for scanning the sample and focusing. The microscope also includes a white light source (LED) for bright field transmission images.

System calibration was performed using the nominal free spectral range of the VIPAs (30GHz) and validated using methanol and water as standards.

### 2.3 Image analysis

Brillouin images were acquired and processed using bespoke software which enables visualization of both white light and raw Brillouin images at each sample position in real time. Any laser drift, and hence changes to the Rayleigh peak positions, will be automatically corrected for. This implementation provides a consistent, user-independent approach to data collection and analysis.

Figure 2

**Fig. 2:**
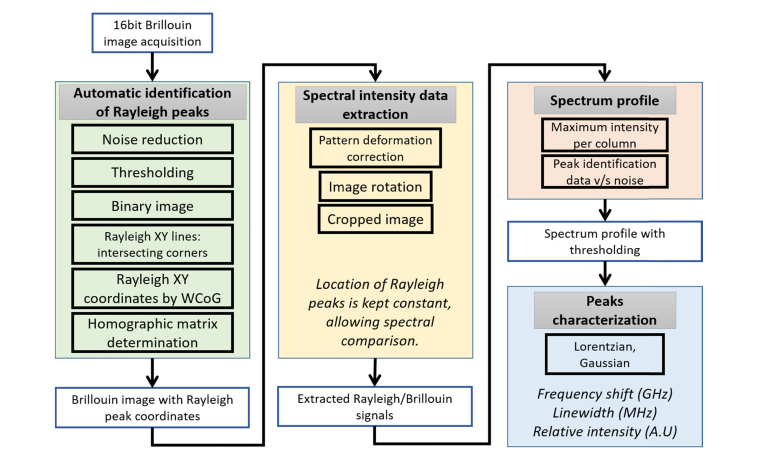
Block diagram showing the stages of the data analysis protocol. The data analysis can be broken down into four major parts (coloured boxes). WCoG denotes weighted centre of gravity.

shows a block diagram of the processing steps that are applied going from a raw image through to finding the Brillouin frequency values and other line shape parameters.

#### Visualization of Brillouin spectra.

**Fig. 3:**
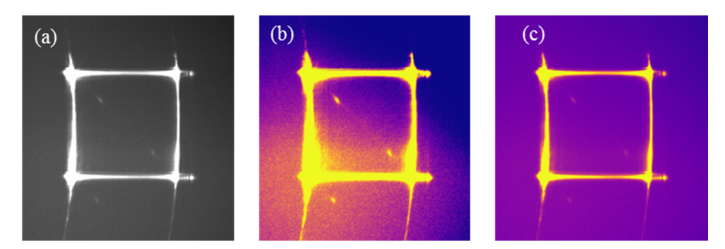
(a) A typical sCMOS output of the spectrometer, showing the Brillouin spectrum of water acquired with a 60x water-immersion objective and 2s exposure time. (b) Corresponding colour image. (c) Contrast-enhanced image.

The first step in image analysis is to improve the visualization of low-contrast images. This operation has no effect on the actual spectral data, however it enables a visual inspection of the output signal. A typical sCMOS output and corresponding processed images are shown in Fig. 3

.

Raw Brillouin images tend to have saturated Rayleigh peaks, as seen from the horizontal and vertical lines in the images, sometimes noisy background and low contrast data, which can make it difficult to visually identify Brillouin signals. To address the need for better visualization of Brillouin spectra, the sCMOS output of the spectrometer is converted into a pseudo colour image (Fig. 3(b)) and then normalised by the standard deviation (Fig. 3(c)), according to:

$$\text{I'=}f\left(I_{o}\right)=\left\{ \begin{array}{l} 0,\;I_{0}<\mu -\alpha \sigma \\ 1,\;I_{0}>\mu +\alpha \sigma \\ \frac{I_{0}-\mu +\alpha \sigma }{2\alpha \sigma },\;\text{otherwise} \end{array}\right.$$ (1)

where $I_{0}$ is the original intensity value, *μ* is the mean of the intensity of the image, *σ* is the standard deviation of the intensities $I_{0}$, and *α* is an arbitrary scaling constant ranging between 0 and 1.I' is a perceptual intensity value, between 0 and 1, which can then be converted into a colour value based on an appropriate colour scale. Note that this enhanced contrast normalization is purely to assist the user and does not take part in the data processing. It is also worth pointing out that in principle Rayleigh peaks can be optically removed from the image, however here they were used to locate the data in the image, and allow for the automated algorithms to find the data regardless of drifts in the image.

#### Identification of Rayleigh peaks.

**Fig. 4:**
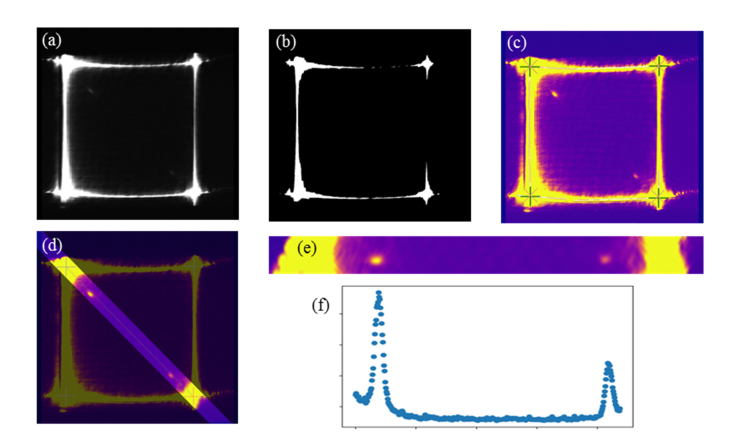
(a) A typical sCMOS output of the spectrometer, showing the Brillouin spectrum of methanol acquired with a 60x water-immersion objective and 2s exposure time. (b) Corresponding denoised thresholded image. (c) Final colour image. Rayleigh peaks are denoted by blue crosses at the corners of the image. (d) Colour image with selected spectral data. (e) Spectral data rotated (by 45° anticlockwise) so that the spectral axis coincides with the x-axis. (f) Brillouin spectrum in which the intensity for each channel corresponds to the maximum intensity recorded by the pixels in each column of the image.

To automatically extract spectroscopic data, the software will first locate the pixels that represent the Rayleigh peaks within the image (Fig. 4

).

The automatic identification of Rayleigh peaks starts by first applying a Gaussian noise filter to the images (Fig. 4(a)) and then a threshold in order to produce a binary image, as illustrated in Fig. 4(b). We chose to use a threshold of 0.95 of the maximum intensity in the image. From this image, the centre pixels of all lines forming the edges of the square can be found (Fig. 4(c)). Using the centre of gravity of this binary image as a guide, the centre pixels can be further related to each corresponding corner. For each corner, a line of best fit is computed for both the horizontal and vertical axes. Here an assumption is made in that the curves arising from the orders of diffraction are approximated as straight lines. Note that this may not be a good approximation if the selected pattern deviates significantly from a square shape. The intersection of these lines gives a good approximation for the position of the Rayleigh peaks. Further refinement of the corner positions is performed by taking an area around each of the intersections and computing the weighted centre of gravity of the original data.

After finding the Rayleigh line coordinates, a homography matrix can be computed and then applied to the image as ‘warp affine’ transformation. This transformation corrects the image by slightly shifting the pixels relative to their original location. By setting the desired location, the data can be transformed to fit a correctly oriented square at a known location and hence to provide a consistent display of imaging data. This also corrects for any displacement in the image due to laser drift or other instabilities of the system that cause distortions to the squared pattern.

#### Extraction of Brillouin spectra.

**Fig. 5:**
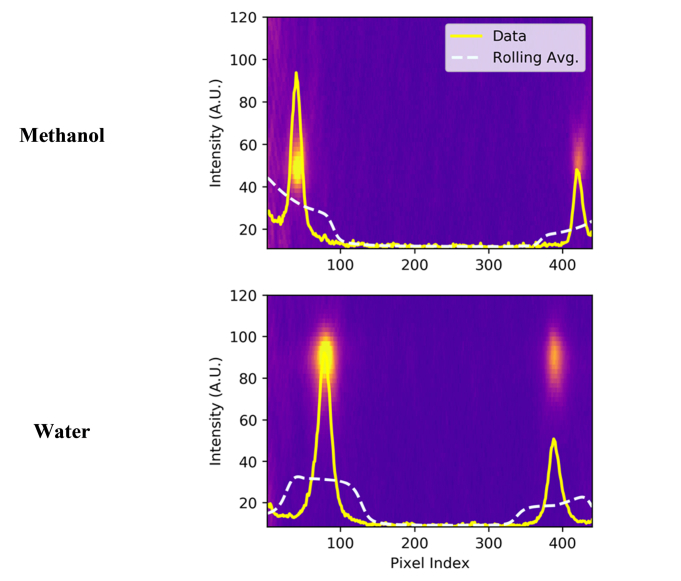
Brillouin spectrum of methanol and water acquired with a 60x water-immersion objective and 2s exposure time. Dashed lines denote lines of average intensity.

Next, the image is cropped and rotated to produce an image containing only the spectroscopic data, as shown in Fig. 4(d) and 4(e). From this, a spectrum is found by taking the maximum intensity recorded by the pixels in each column of the image, and this value is retained as the intensity for each channel of the Brillouin spectrum (Fig. 4(f)). The location of the Rayleigh peaks in these images is consistent, which allows for spectra to be directly compared, as shown in Fig. 5

.

Setting the distance between Brillouin peaks equal to the nominal FSR of the spectrometer, it is possible to convert the dispersion axis from a pixel index scale to a frequency scale (GHz) through the expression:

$$\nu_{B}=\frac{\text{FSR}\cdot \text{min}\left(i-R_{0},R_{1}-i\right)}{R_{1}-R_{0}}$$ (2)

where ν*_Β_* is the Brillouin frequency shift, *R*_0_ and *R*_1_ are the pixel positions of the Rayleigh peaks, and *i* is the pixel index.

Separating data from the background noise is achieved by comparing the intensity at a given pixel index with the average of the surrounding intensities. This is illustrated in Fig. 5. Adjacent points above this threshold are considered to be part of the same peak. Apart from preselecting an appropriate threshold, the process is fully automated.

In this system, a linear relationship between frequency and position of the lines was adequate to achieve results comparable to those previously published (see below). In future work, we will investigate a nonlinear calibration to generalize the approach further.

#### Brillouin peak analysis.

**Fig. 6:**
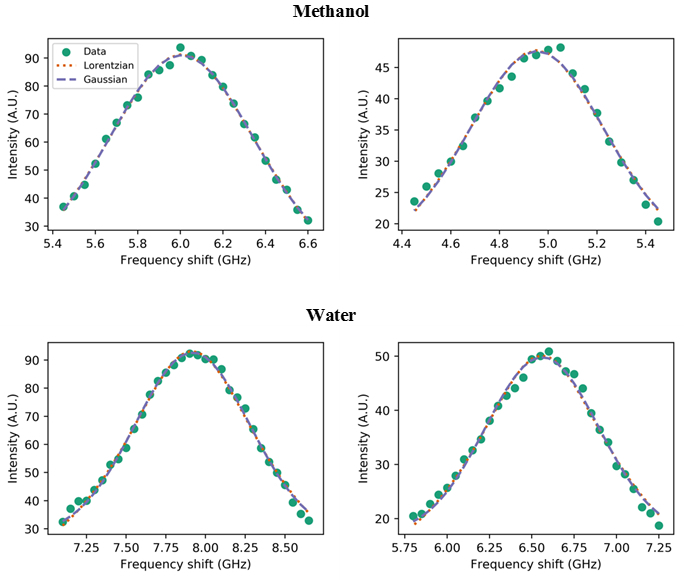
Fit results for (left) Stokes and (right) anti-Stokes parts of the Brillouin spectrum of methanol and water. Lorentzian and Gaussian functions were used resulting in *R*^2^ = 0.996 and 0.982 (methanol), 0.994 and 0.989 (water), respectively. Note that negative frequency shifts have been converted to positive shifts.

Once the relevant peaks have been identified, it is possible to proceed with line shape analysis. Various function types can be implemented for curve fitting of the Brillouin peaks. Here we used Lorentzian functions and nonlinear least squares fitting, and compared the results with those from a Gaussian fit. Figure 6

illustrates the fit results for both Stokes and anti-Stokes peaks of methanol and water.

In addition to the peak parameters, the fitting algorithm provides the root mean square error (RMS) and the regression coefficient (*R*^2^) which are used to assess the quality of the fit. The fit results show that a Lorentzian fit works better than a Gaussian fit, but only marginally, owing to convolution with the instrumental function.

## 3. Results and discussion

We validated the accuracy of spectral axis calibration using methanol [18] and water [4] data from the literature, listed in Table 1

**Table 1:** Brillouin frequency shift and linewidth for methanol and water. *^a^* Data from ref [18]. *^b^* Data derived using a high-resolution tandem Fabry-Pérot interferometer [4].

**Sample**	**Wavelength (nm)**	**Frequency shift (GHz)**		**Linewidth (GHz)**	

Methanol	532	5.6*^a^*	5.5	-	0.7
Water		7.5*^b^*	7.3	0.65*^b^*	0.9

.

We notice a close correspondence between our data and those from the literature, indicating that the method can now be applied for the characterization of samples of interest.

Figure 7

**Fig. 7:**
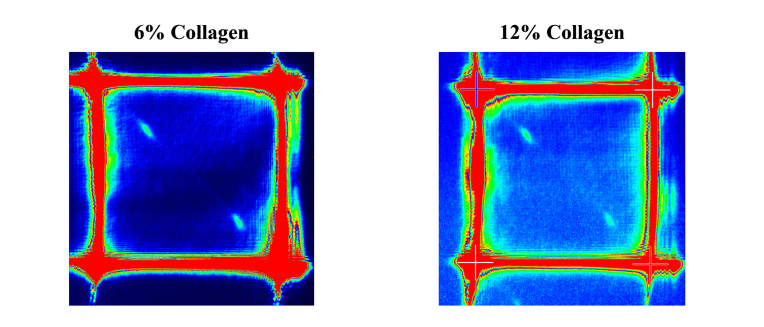
Brillouin spectrum of collagen gelatins with 6 and 12 wt. % collagen acquired with a 60x water-immersion objective, and 3s and 2s exposure time, respectively.

shows typical Brillouin images of collagen gelatins at two protein concentrations.

**Fig. 8:**
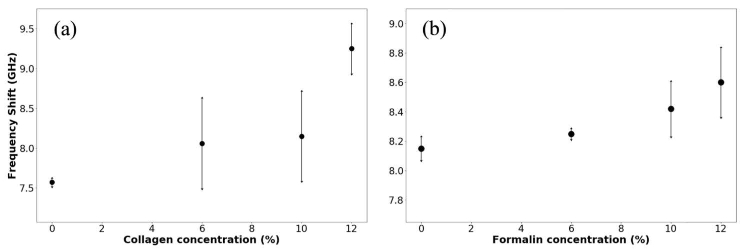
Plot of frequency shift vs. (a) collagen concentration and (b) formalin concentration at 10 wt. % collagen concentration for the gelatin hydrogels.

Fitting results for these data are plotted in Fig. 8

.

Here, we note an increase in frequency shift of the Brillouin peak upon increase of collagen concentration (Fig. 8(a)) and upon addition of formalin at constant protein concentration (Fig. 8(b)). The frequency shifts obtained here are consistent with those previously reported for similar gels [19]. From the data in Fig. 8, longitudinal elastic moduli ranging between *ca.* 2 and 3 GPa were estimated.

These results suggest that increasing collagen or formalin concentration leads to elevated stiffness of the gel, and that the effect is less pronounced in formalin-containing gels plausibly due to a different gel structure.

## 4. Conclusion

In summary, new software was developed for VIPA Brillouin image analysis to improve visualization and extraction of spectral data in real time. This was validated on standard liquids and applied to collagen gelatin hydrogels, both in the presence and absence of a cross-linker. We have shown that we can obtain accurate spectral data from tissue phantoms using a simplified optical system and bespoke software algorithms.

The results show that the Brillouin frequency shift increases with increasing collagen or formalin content, which suggests an increase in stiffness as the water content is reduced. This work demonstrates the ability of this method to determine the Brillouin spectral signatures of biologically relevant specimens close to physiological hydration levels.
